# Supplementary material for: Observation of boundary induced chiral anomaly bulk states and their transport properties
Source: Nat Commun. 2022 Oct 7;13:5916. doi: 10.1038/s41467-022-33447-x (PMC9546894; doi:10.1038/s41467-022-33447-x)
Supplement: Supplementary file 1 — Supplementary Information [file 41467_2022_33447_MOESM1_ESM.pdf]

Supplementary Information for

Observation of boundary induced chiral anomaly bulk states and their transport properties

Mudi Wang[1], Qiyun Ma[2], Shan Liu[2], Ruo-Yang Zhang[1], Lei Zhang[3,4], Manzhu Ke[2], Zhengyou Liu[2,5*], and C. T. Chan[1*]

[1]Department of Physics, The Hong Kong University of Science and Technology, Hong Kong, China

[2]Key Laboratory of Artificial Micro- and Nanostructures of Ministry of Education and School of Physics and Technology, Wuhan University, Wuhan, China

[3]State Key Laboratory of Quantum Optics and Quantum Optics Devices, Institute of Laser Spectroscopy, Shanxi University, Taiyuan 030006, China

[4]Collaborative Innovation Center of Extreme Optics, Shanxi University, Taiyuan 030006, China

[5]Institute for Advanced Studies, Wuhan University, Wuhan, China

*Correspondence to: zyliu@whu.edu.cn; phchan@ust.hk

This file includes:

Supplementary Text

Figs. S1 to S11

Supplementary Text

Contents

1 The band dispersion of the PC slab with different layer numbers (integer).

2 The theoretical explanation and simulation results on the influence of the boundary conditions.

3 Experimental Fourier spectra for more frequencies in bend and disordered PCs.

4 Details of the modified waveguide samples.

5 The influence about the different cutting boundaries.

6 The CABSs in electromagnetic wave systems.

1 The band dispersion of the PC slab with different layer numbers (integer)

Figure S1 shows the evolution of the CABS for PC strips with different layer numbers, from a minimum of 2 layers to a wide strip. If the width of PC remains an even (or odd) number layer, the dispersion stays the same, while the group velocities switch their signs when the layer thickness number changes from even to odd. We note that the frequency range of the CABSs is reduced with increasing width of the PC waveguide. In the limit of a very wide waveguide, the pseudo-gap with gradually shrink and the waveguide dispersion is equivalent to the projected band structure of the bulk phononic crystal, but the chiral anomaly remains at the Dirac point as long as the slab is of finite thickness and bounded by hard-wall boundary conditions.

2 The theoretical explanation and simulation results on the influence of the boundary conditions.

Here we use a boundary matrix method [see for example, Ref. 28, 29] to analytically solve the wavefunctions and dispersions of all the states near the K point (the case in K' point is similar, since the system has time-reversal symmetry).

The physics near the K valley is effectively characterized by the 2D Dirac Hamiltonian:

$$H = \sigma_x k_x v_D - i\partial_y \sigma_y v_D, \tag{S1}$$

where k_x denotes the parallel wavevector relative to the K point. The bulk eigenstates at the K valleysatisfy the static 2D Dirac equation

$$H|\varphi(k_x, y)\rangle = \varepsilon|\varphi(k_x, y)\rangle. \tag{S2}$$

Since the system has rigid boundaries at $y = 0$ and $y = L$, the wave function bounded by the rigid boundaries can be expressed as the superposition of the two linearly independent bulk eigenstates at each k_x:

$$|\varphi(k_x, y)\rangle = \alpha_1|\varphi_1(k_x, y)\rangle + \alpha_2|\varphi_2(k_x, y)\rangle \quad (0 \le y \le L), \tag{S3}$$

where the two bulk eigenstates $|\varphi_1(k_x, y)\rangle = \begin{pmatrix} (k_x - u)v_D \\ \varepsilon \end{pmatrix} \exp(uy)$ and $|\varphi_2(k_x, y)\rangle = \begin{pmatrix} (k_x + u)v_D \\ \varepsilon \end{pmatrix} \exp\big(-u(y - L)\big)$ with $u = \sqrt{k_x^2 - \varepsilon^2/v_D^2}$ are solved from Eq. (S2).

Hence, we can get

$$|\varphi(k_x,y)\rangle = \alpha_1\cdot\begin{pmatrix}(k_x-u)v_D\\ \varepsilon\end{pmatrix}\cdot\exp(uy)+\alpha_2\cdot\begin{pmatrix}(k_x+u)v_D\\ \varepsilon\end{pmatrix}\cdot\exp\big(-u(y-L)\big)$$

$$=\begin{pmatrix}(k_x-u)v_D\exp(uy) & (k_x+u)v_D\exp\big(-u(y-L)\big)\\ \varepsilon\exp(uy) & \varepsilon\exp\big(-u(y-L)\big)\end{pmatrix}\begin{pmatrix}\alpha_1\\ \alpha_2\end{pmatrix}\ (0\le y\le L) \tag{S4}$$

Next we will examine the effect of bulk state when changing boundaries. The effect of the boundary tuning at $y=0$ and $y=L$ can be represented as

$$\widehat{\mathrm{M}}_1|\varphi(k_x,0)\rangle=|\varphi(k_x,0)\rangle, \tag{S5}$$

$$\widehat{\mathrm{M}}_2|\varphi(k_x,L)\rangle=|\varphi(k_x,L)\rangle. \tag{S6}$$

Here $\widehat{\mathrm{M}}_1$ and $\widehat{\mathrm{M}}_2$ are 2×2 unitary matrix satisfying $\widehat{\mathrm{M}}^2=1$. Two rigid boundaries makes that the bulk states can only propagate along the *x*-direction, while the *y*-direction is forbidden, hence $\{\widehat{\mathrm{M}},\sigma_y\}=0$ [Ref. 29].

By virtue of these constraints, the two the boundary matrixes $\widehat{\mathrm{M}}_1$ and $\widehat{\mathrm{M}}_2$ can be expressed as

$$\widehat{\mathrm{M}}_1(\theta_1)=\sigma_x\sin\theta_1+\sigma_z\cos\theta_1, \tag{S7}$$

$$\widehat{\mathrm{M}}_2(\theta_2)=\sigma_x\sin\theta_2+\sigma_z\cos\theta_2, \tag{S8}$$

where θ_1 and θ_2 are determined by the lower (upper) boundary condition of the real system.

The wavefunction $|\varphi(k_x,y)\rangle$ at $y=0$ and $y=L$ should satisfy Eqs. (S5, S7) and Eqs. (S6, S8) respectively, which indicates $|\varphi(k_x,0)\rangle$ and $|\varphi(k_x,L)\rangle$ are the eigenstates of $\widehat{M}_1$ and $\widehat{M}_2$ corresponding to the eigenvalue of $+1$, respectively:

$$|\varphi(k_x,0)\rangle = \beta_1\begin{pmatrix}\sin\theta_1\\ 1-\cos\theta_1\end{pmatrix}, \tag{S9}$$

$$|\varphi(k_x,L)\rangle = \beta_2\begin{pmatrix}\sin\theta_2\\ 1-\cos\theta_2\end{pmatrix}. \tag{S10}$$

There are four unknown parameters α_1, α_2, β_1, β_2 for four linear Eqs. (S4, S9, S10).

$$R\begin{pmatrix}\alpha_1\\ \alpha_2\\ \beta_1\\ \beta_2\end{pmatrix}=\begin{pmatrix}(k_x-u)v_D & (k_x+u)v_De^{uL} & -\sin\theta_1 & 0\\ \varepsilon & \varepsilon e^{uL} & -1+\cos\theta_1 & 0\\ (k_x-u)v_De^{uL} & (k_x+u)v_D & 0 & -\sin\theta_2\\ \varepsilon e^{uL} & \varepsilon & 0 & -1+\cos\theta_2\end{pmatrix}\begin{pmatrix}\alpha_1\\ \alpha_2\\ \beta_1\\ \beta_2\end{pmatrix}=\begin{pmatrix}0\\ 0\\ 0\\ 0\end{pmatrix} \tag{S11}$$

This equation has nonzero solutions when the determinant of the matrix R vanishes:

$$\det R(\varepsilon, k_x, \theta_1, \theta_2) = 0. \tag{S12}$$

For a fixed boundary condition, θ_1 and θ_2 are determined, and only ε and k_x is unknown in Eqs. (S11) (This equation is hard to get analytical solution, but their relationship is deterministic). Hence, the relationship between ε and k_x can be obtained:

$$\varepsilon = f(k_x, \theta_1, \theta_2). \tag{S13}$$

Especially, when $\theta_1 = \theta_2 = \frac{\pi}{2}$, we have $\widehat{M}_1 = \widehat{M}_2 = \sigma_x$ is coincident with the mirror-y operator of the Dirac Hamiltonian (see main text) and $|\varphi(k_x, 0)\rangle = |\varphi(k_x, L)\rangle \propto \begin{pmatrix} 1 \\ 1 \end{pmatrix}$ indicates the boundary conditions select the bulk even modes with $\varepsilon = k_x v_D$, which corresponds to the band structure in Fig. 1c. The existence of boundaries makes additional boundary potential to be applied in the vicinity of the boundaries. We take the additional boundary potential $V_1(y)$ applied in the vicinity of the bottom edge ($V_1(0 < y < y_0) \neq 0$ and $V_1(y \geq y_0) = 0$ with $y_0 \to 0$) as an example.

The modified Hamiltonian is

$$H = \sigma_x k_x v_D - i\partial_y \sigma_y v_D + V_1(y). \tag{S14}$$

The transfer matrix $\mathrm{T}(y_1, y_2)$ are introduced to connect the wavefunctions $|\varphi_k(y)\rangle$ from y_1 to y_2.

$$|\varphi(k_x, y_1)\rangle = \mathrm{T}(y_1, y_2)|\varphi(k_x, y_2)\rangle, \quad 0 < y_2 < y_1 < L, \tag{S15}$$

According to Eqs. (S2, S14, S15), the transfer matrix $\mathrm{T}(y_1, y_2)$ can be obtained:

$$T(y_1, y_2) = \mathrm{Pexp} \int_{y_2}^{y_1} \frac{1}{v_D} (\sigma_z k_x - iV_1(y)\sigma_y) \cdot dy, \tag{S16}$$

where Pexp is a path-ordered exponential.

If no boundary potential is applied, the transfer matrix is T_0. When $y \geq y_0$, the transfer matrix can be expressed as

$$T(y, 0) = T(y, y_0)T(y_0, 0) = T_0(y, y_0)T(y_0, 0), \ (y \geq y_0), \tag{S17}$$

and the bulk states can be written as

$$|\varphi(k_x, y \geq y_0)\rangle = \mathrm{T}(y, y_0)|\varphi(k_x, y_0)\rangle. \tag{S18}$$

According to Eqs. (S5, S18), the following relationship is established

$$[T(y,0)\widehat{M}_1 T^{-1}(y,0)]|\varphi(k_x,y_0)>=|\varphi(k_x,y_0)>. \tag{S19}$$

$\widehat{M}_{1eff}$ is the equivalent boundary matrix and satisfy

$$\widehat{M}_{1eff}|\varphi(k_x,y_0)>=|\varphi(k_x,y_0)>. \tag{S20}$$

Comparing Eqs. S19 and S20, we can get

$$\widehat{M}_{1eff}=T(y,y_0)\cdot\widehat{M}_1(\theta_1)\cdot T^{-1}(y,y_0). \tag{S21}$$

Since y_0 is very small compared with wavelength, the Eq. (S16) can be simplified to

$$T(y_0,0)=\exp(i\theta_{V_1}\sigma_y),\ \theta_{V_1}=-\int_0^{y_0}\frac{V_1(y)}{v_D}dy. \tag{S22}$$

Combining Eqs. (S21) and (S22), one obtains a simple form of the effective boundary matrix which is identical to the original boundary matrix up to a parameter shift θ_{V1}:

$$\widehat{M}_{1eff}=\widehat{M}_1(\theta_1+\theta_{V1}). \tag{S23}$$

The additional boundary potential $V_2(y)$ applied in the vicinity of the upper edge $(y\to L)$ has the similar effect:

$$\widehat{M}_{2eff}=\widehat{M}_2(\theta_2+\theta_{V2}). \tag{S24}$$

These verify that modifying the boundary matrix can simulate the effect of adding additional boundaries. Hence when two additional boundary potentials $V_1(y)$ and $V_2(y)$ are added, the energy ε will the following change:

$$\varepsilon=f(k_x,\theta_1+\theta_{V1},\theta_2+\theta_{V2}). \tag{S25}$$

Moreover, we show the influence of the boundary truncation positions on the band structure (simulated by COMSOL) in Fig. S2. In the process of changing the boundary truncation positions at two outmost layers, we ensure that the PC waveguide maintains mirror-*y* symmetry. Fig. S2a shows the band structure of the PC slab with a width varying from 10 to 12 layers (normalized by $\frac{\sqrt{3}}{2}a$) by shifting the truncation positions. We focus on the frequencies near 15.5 kHz, which are in the frequency range of the CABSs. When the width of the PC slab increases from 10 to 12 layers, the band structure changes continuously, and no band gap appears. Compared to the CABSs for the PC with 10 layers, the slope of the CABS is reversed at each valley when the layer number increases to 11, while the dispersion is restored when the layer

number is 12. These results show that shifting the truncation position one lattice constant at each boundary represents a complete change cycle for the CABSs, therefore, the band structure can be adjusted conveniently by adjusting the boundary truncation positions. The change in the group velocity of the CABS as a function of the PC width at approximately 15.5 kHz is shown in Fig. S2b. Compared to the group velocity when the layer number is 10, the CABS group velocity is reversed when the layer number is 11, while it is restored when the layer number is 12. These results are consistent with the change in the band structure.

3 Experimental Fourier spectra for more frequencies in bend and disordered PCs

Fig. S3 shows that for the frequencies 14.9, 15.2, 15.8 and 16.1 kHz in the CABS region, a sharp bend and disorder cannot destroy the single-valley locked transport. This experimentally verifies the robust properties of CABSs over a wide frequency range inside the pseudo-gap.

4 Details of the modified waveguide samples

The structural details of the modified waveguides in Fig. 4b,c are shown in Fig. S5a,b respectively.

5 The influence about the different cutting boundaries

The 60-bend waveguide, as shown in Fig. 4a, can be seen as consisting of two waveguides in domain 1 and 3, and the junction of the two waveguides is the coupling region. The sound wave cannot pass through the 60-bend waveguide smoothly. In order to change this state, we need to make some modifications in the coupling region. Since two waveguides (domain 1 and 3) are symmetric about the middle line of the coupler (i.e. the line connecting the two corners). To maintain the symmetry, we truncate the coupling region along an angle of 30 degrees from the *x*-direction. At the same time, we know that the transmittance of the waveguide is related to its width. Therefore, under the premise of ensuring symmetry, we cut different widths in

coupling region, as shown in Fig. S6a, to see which one has the highest transmission. And we find the truncated boundary 2 has the best results, the transmission of the 60-bend waveguide (green color in Fig. S6b) closes to 1 in the shaded frequency range. As a result, the sound wave can pass through the 60 degree-bend in Fig. 4c, and the reflection is highly suppressed (which is proved by the Fourier transformation in domain 1 where only K valley states are excited), after the boundary modification.

6 The CABSs in electromagnetic wave systems

The concept of creating CABSs by modifying the boundary in acoustic systems can also be applied to other systems. Here, we give two examples based on electromagnetic wave systems in microwave frequency and light wave frequency, respectively.

In microwave frequency, we consider a hexagonal dielectric photonic crystal (16.0 mm lattice constant) of circular rods (4.0 mm diameters) arranged in a hexagonal lattice in an air background, as shown in Fig. S7. The rods have permittivity $\varepsilon = 12$ and permeability $\mu = 1$. The bulk dispersion (right panel) shows the expected Dirac cone dispersion, with the 2nd and 3rd band crossing at the K and K’ points. The modes along ΓK are can be classified as even/odd (middle panel) according to the mirror plane indicated by the dotted line in the unit cell (left panel). For a waveguide of finite width, a pseudo-gap will appear at the K/K’ point, with the size of the gap depending on the width, as in the case of acoustics. A chiral anomaly (a one-way mode in one valley) will appear in the pseudo-gap due to the boundary condition, as shown in the text.

For light wave frequencies, we consider a dielectric slab with a hexagonal array of air holes (150 nm lattice constant and 120 nm diameters) in air, as shown in Fig. S8a. Open boundary conditions were applied at the top and bottom boundary of the waveguide, while the period boundary conditions were assumed at left and right, as shown in Fig. S8b. The material parameters of the dielectric slab are represented by the permittivity $\varepsilon = 12$ and permeability $\mu = 1$. The wave guiding phenomenon in the light wave frequency is similar to the phenomenon in the acoustic system and

microwave frequencies. This is because the interface between the slab with a high dielectric constant ($\varepsilon = 12$) and the air has similar effect to the hard boundary in acoustic system.

These phenomena show that it is also feasible to generate CABSs by modifying the boundary in electromagnetic wave systems.

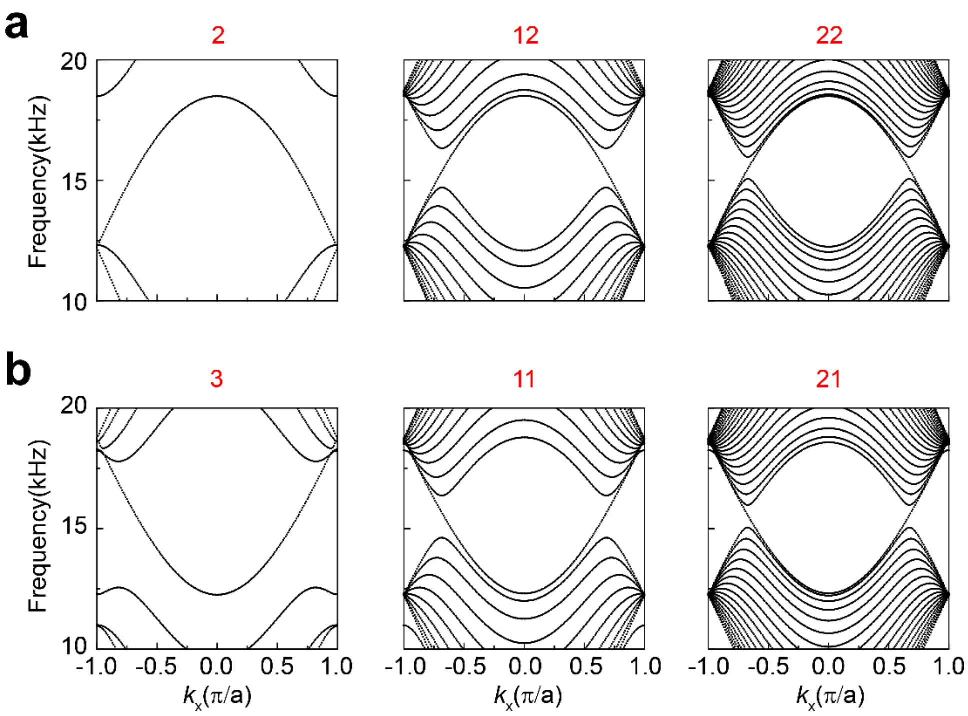

Fig. S1. The change in the band dispersion of the PC with the increasing layer number (normalized by $\frac{\sqrt{3}}{2}a$). **a,** All the layers are the even numbers. **b,** All the layers are the odd numbers.

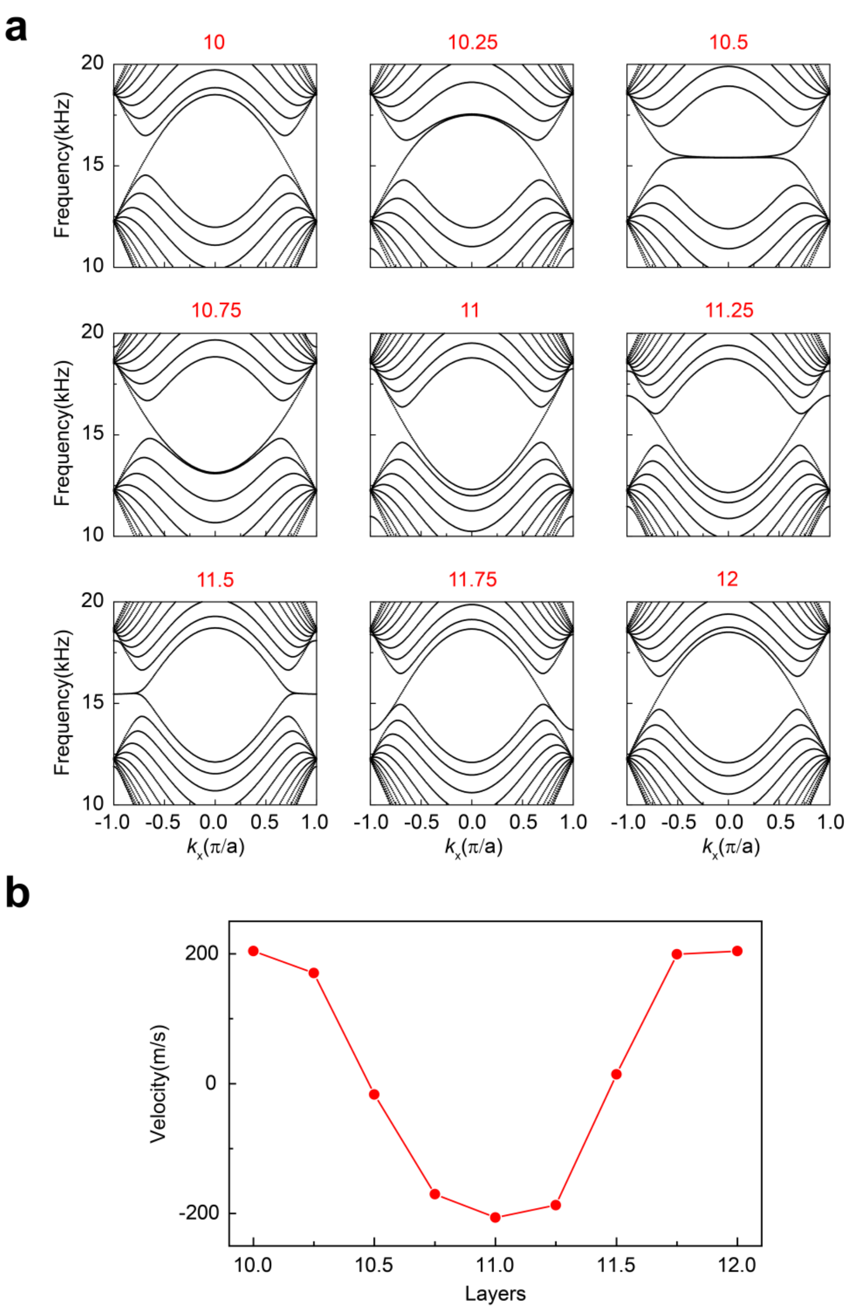

Fig. S2. a, The change in band dispersion of the PC with increasing slab layers. **b,** The change in the group velocities of the CABS near 15.5 kHz as a function of layer number.

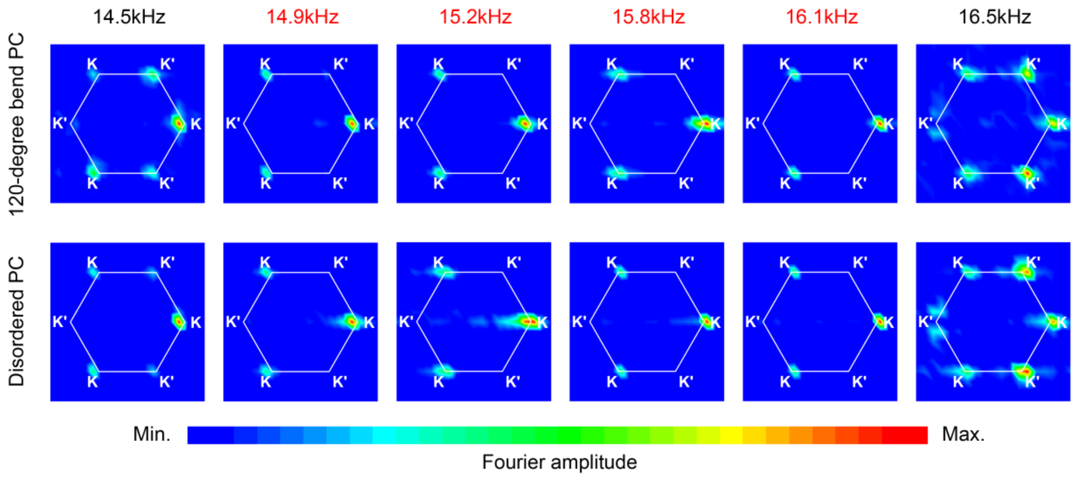

Fig. S3. Experimental Fourier spectra of the acoustic field in the magenta dashed rectangle in **Fig. 3a** (120-degree-bend PC) and **Fig. 3b** (disordered PC) for different frequencies, while the region containing CABSs spans from 14.5 kHz to 16.5 kHz.

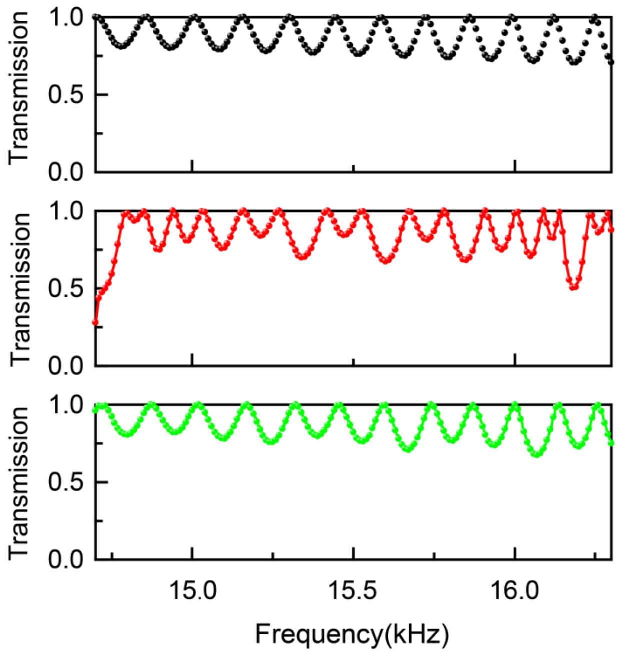

Fig. S4. The transmissions of the straight waveguide without defects, 120-degree-bend waveguide and the disordered waveguide at the frequency of CABSs.

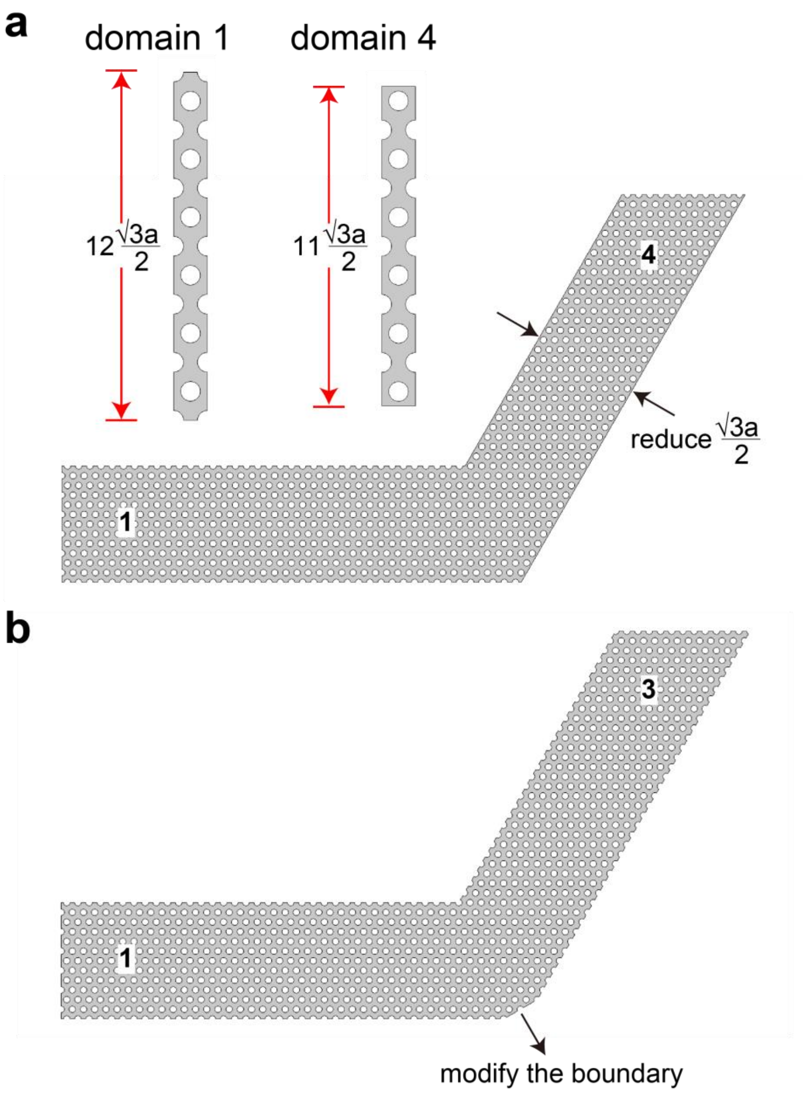

Fig. S5. Diagrams showing the boundary modifications to form the models in **Fig. 4b** and Fig. **4c**.

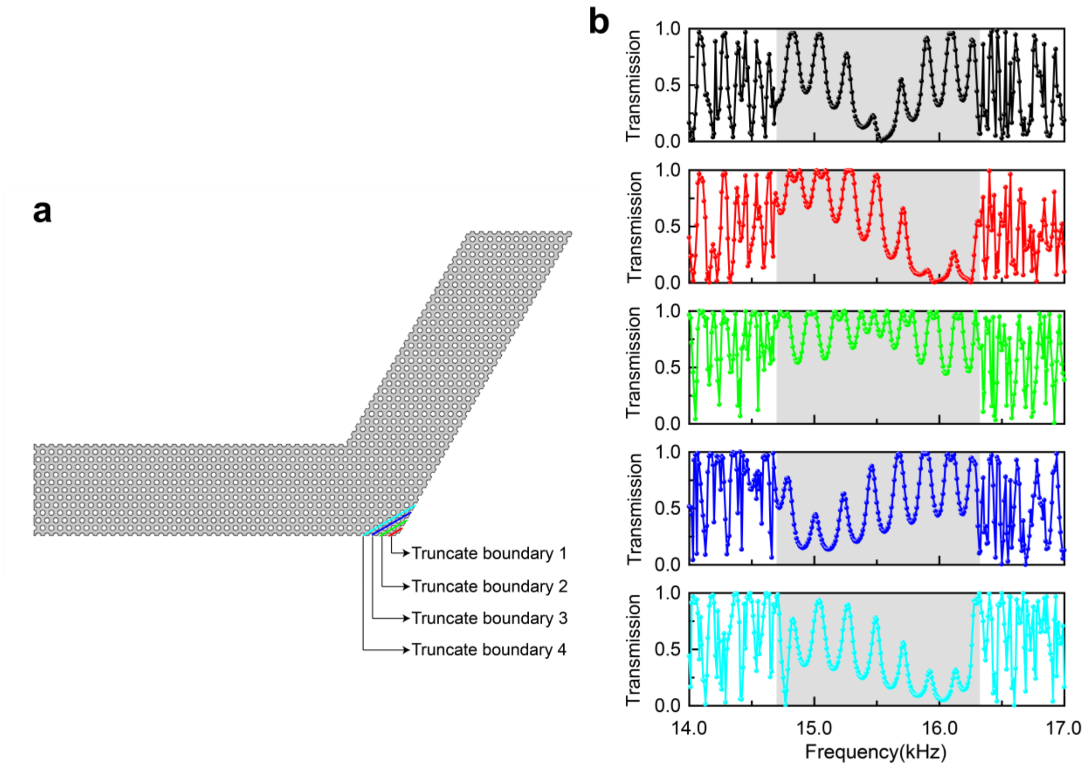

Fig. S6. a, Diagrams showing four different truncated boundaries with different colors at the joint region for the 60-degree waveguide. **b**, From top to bottom are the transmissions of unmodified waveguide and four waveguides with truncated boundaries 1-4.

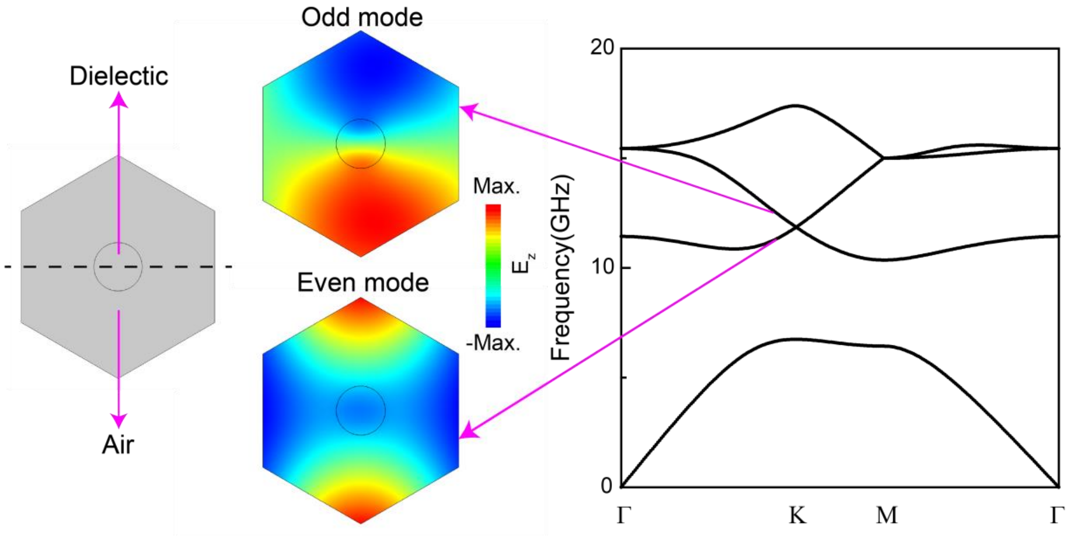

Fig. S7. Schematic of the unit cell of photonic crystal (for microwave frequency). Right panels: Bulk band diagram and the corresponding electric field (colors) of the eigen states in the unit cell.

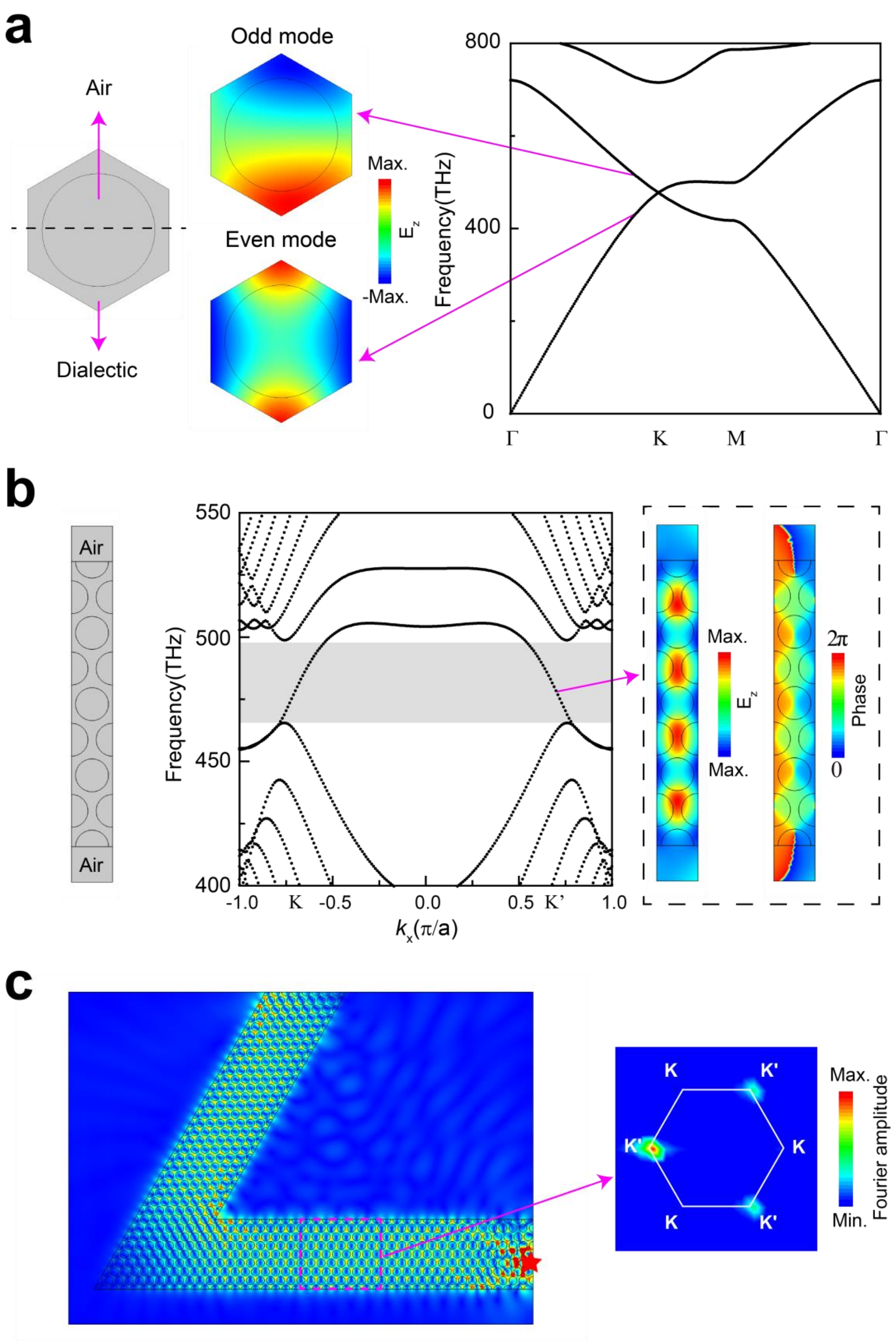

Fig. S8. a, Schematic of the unit cell of photonic crystal (for light wave frequency). Right panels: Bulk band diagram and the corresponding electric field (colors) of the eigen states in the unit cell. **b,** Photonic crystal waveguide

in air with open boundary condition above and below and periodic boundary condition on the left and right. Right panel: band dispersion of the photonic crystal slab and the corresponding electric field (colors) and the phase (colors) field of the CABSs (shadow region). **c,** Simulated electric field distributions at 483 THz for a point source (red star) on the right of the 120-degree-bend photonic crystal. Right panels: the corresponding theoretical Fourier spectra of the electric fields in the magenta dashed rectangle.

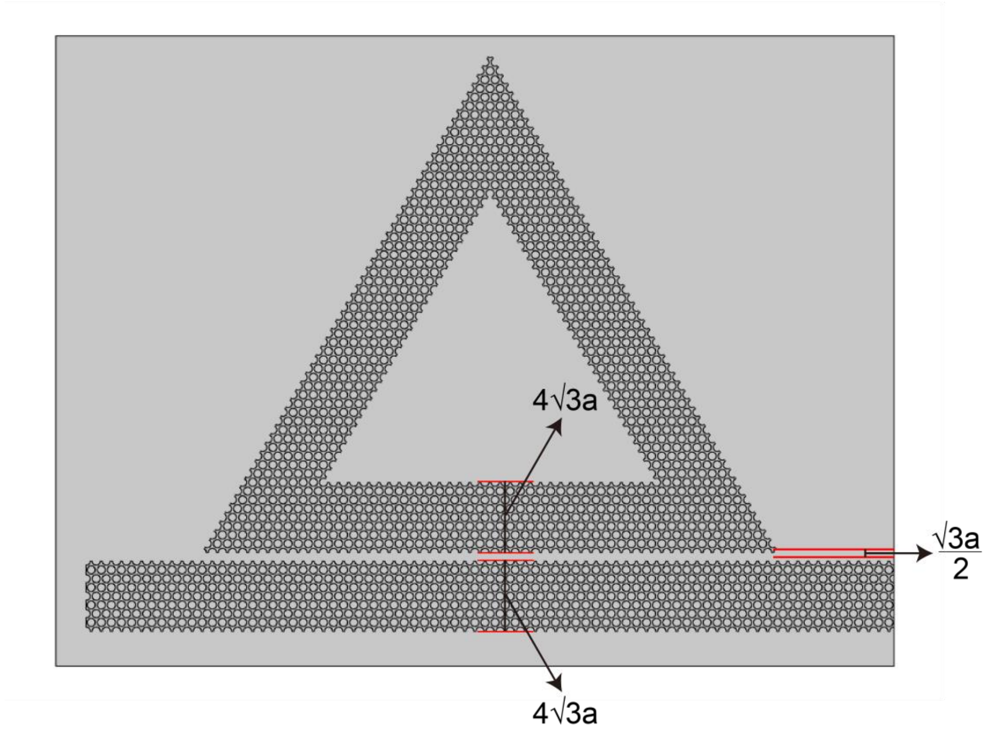

Fig. S9. Diagram of the waveguide and the resonator.

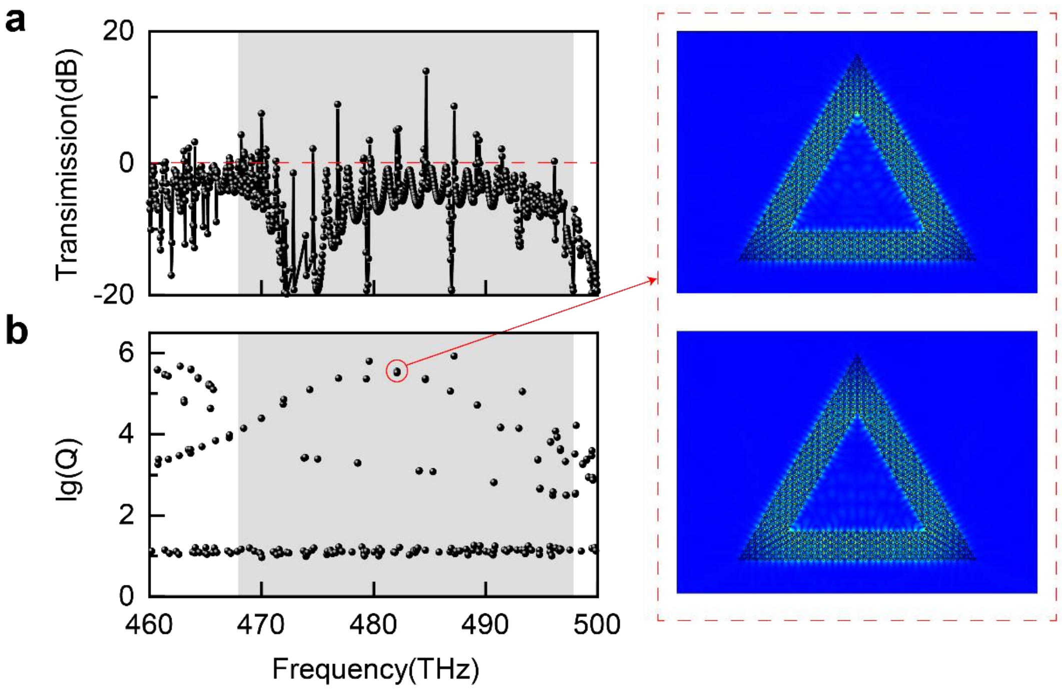

Fig. S10. a, The simulation transmission spectra of the straight waveguide in Fig. 6c. The dielectric constant in the triangular resonator is $12 + 0.002 * i$. The straight waveguide has no gain incorporated. The small gain in the triangle resonator results in amplified transmission at some resonant frequencies. **b**, Calculated eigenmode Q factor (lg(Q)) for the resonator in Fig. 6c. Right panel: the two eigenmode at 482 THz.

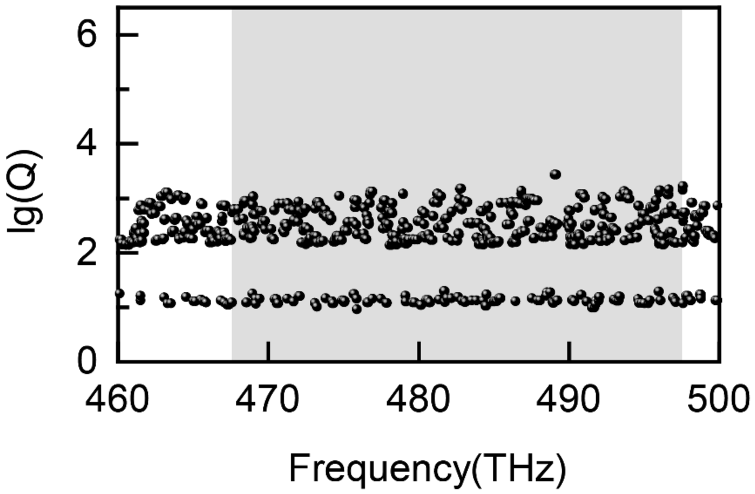

Fig. S11. a, Calculated eigenmode Q factor (lg(Q)) for the solid triangle in Fig. 6d.
